# Supplementary material for: Retinoic acid induces human gastruloids with posterior embryo-like structures
Source: Nat Cell Biol. 2024 Aug 20;26(10):1790–803. doi: 10.1038/s41556-024-01487-8 (PMC11469962; doi:10.1038/s41556-024-01487-8)
Supplement: Supplementary file 1 — Supplementary Figs. 1–21. [file 41556_2024_1487_MOESM1_ESM.pdf]

nature cell biology

Supplementary information

https://doi.org/10.1038/s41556-024-01487-8

Retinoic acid induces human gastruloids with posterior embryo-like structures

In the format provided by the authors and unedited

Supplementary Figures

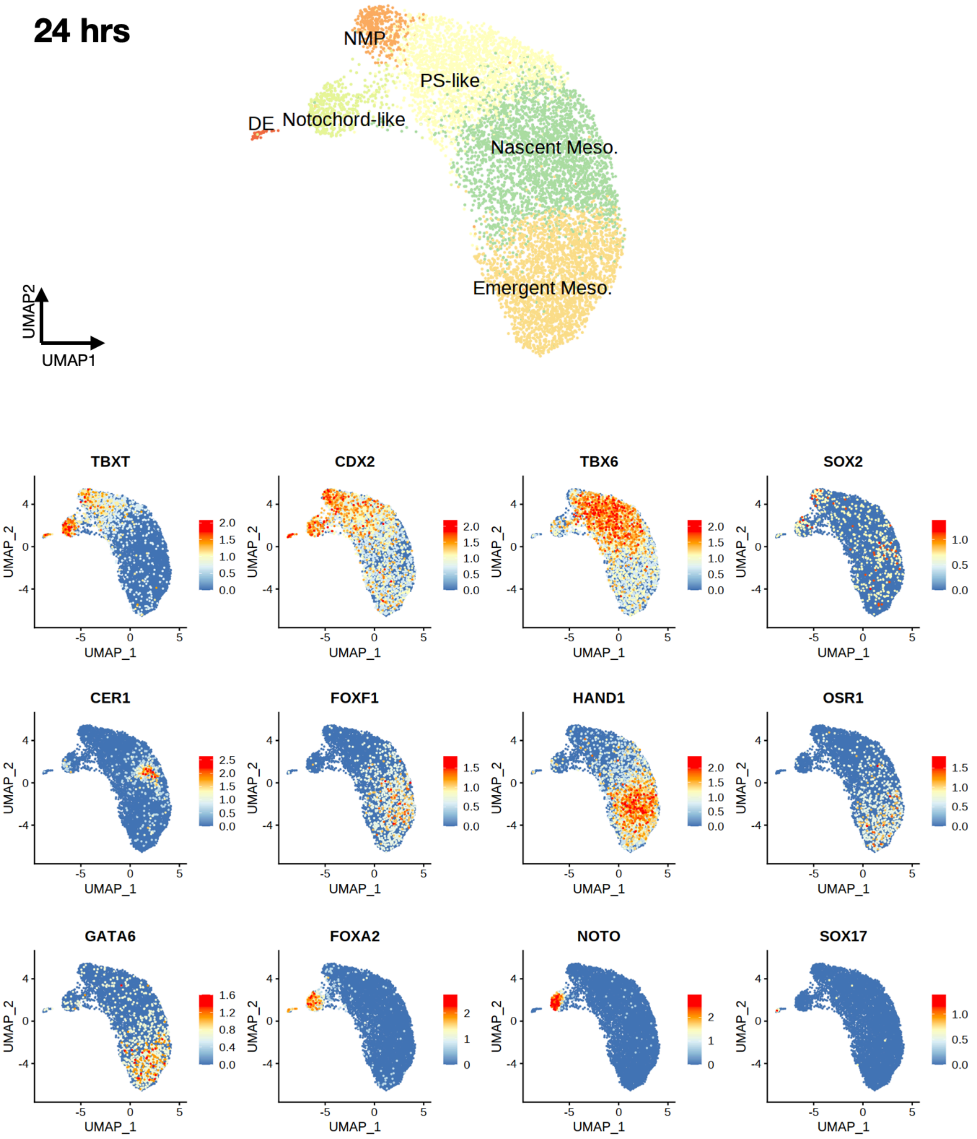

Supplementary Fig.1. Marker gene expression & cell type annotation in human gastruloids at 24 hrs after induction.

UMAP with unsupervised clustering of scRNA-seq profiles is shown, along with expression of marker genes used to annotate these clusters as a continuum of primitive streak-like, nascent mesoderm and emergent mesoderm, along with notochord-like cells and definitive endoderm. DE, definitive endoderm; PS, primitive streak.

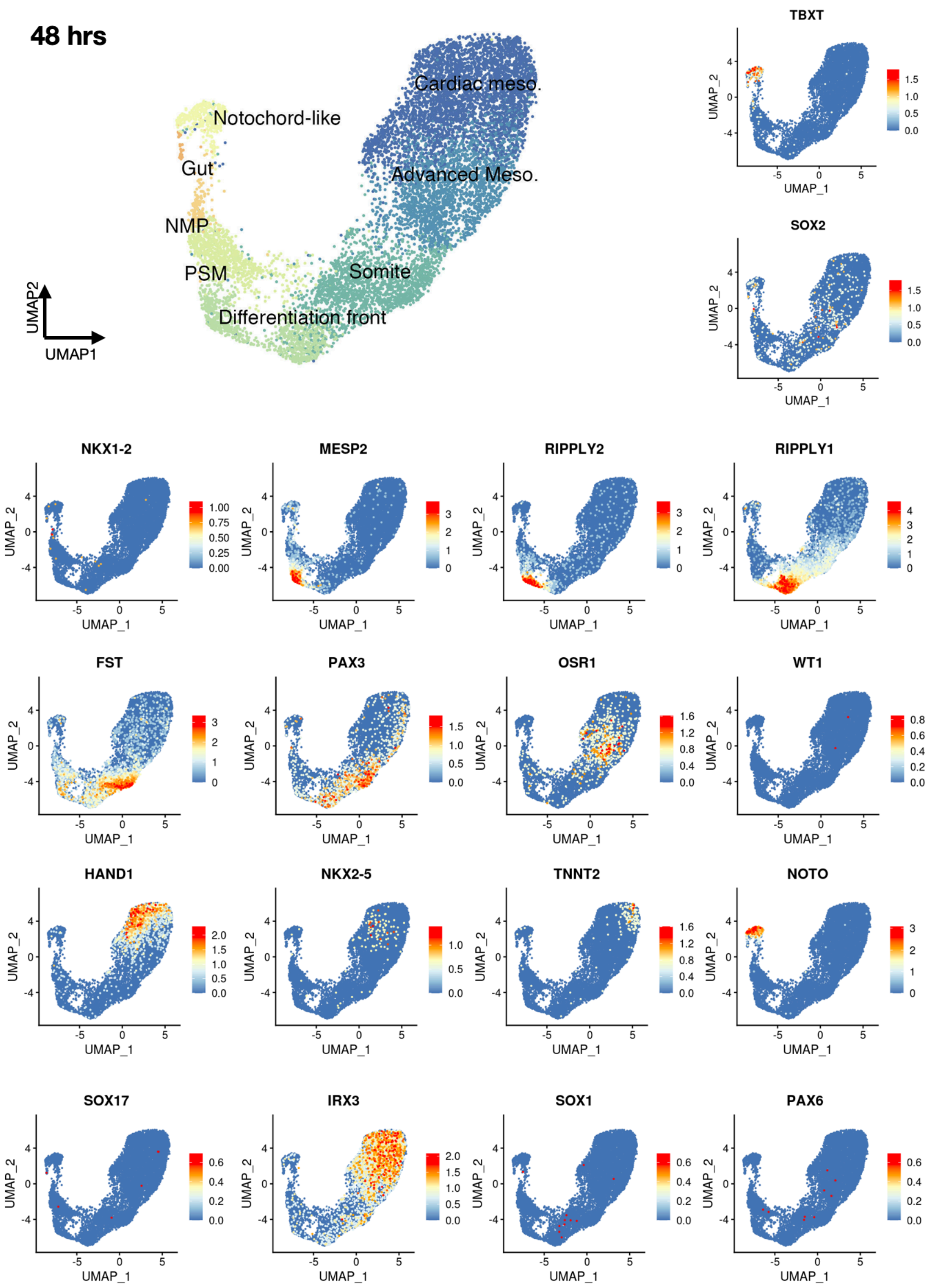

Supplementary Fig.2. Marker gene expression & cell type annotation in human gastruloids at 48 hrs after induction.

UMAP with unsupervised clustering of scRNA-seq profiles is shown, along with expression of marker genes used to annotate these clusters as a continuum of neuromesodermal progenitors, presomitic mesoderm, differentiation front, and somites; additionally, advanced mesoderm and cardiac mesoderm, as well as notochord-like and gut/endoderm-like cells, were detected. NMP, neural mesodermal progenitor; PSM, presomitic mesoderm.

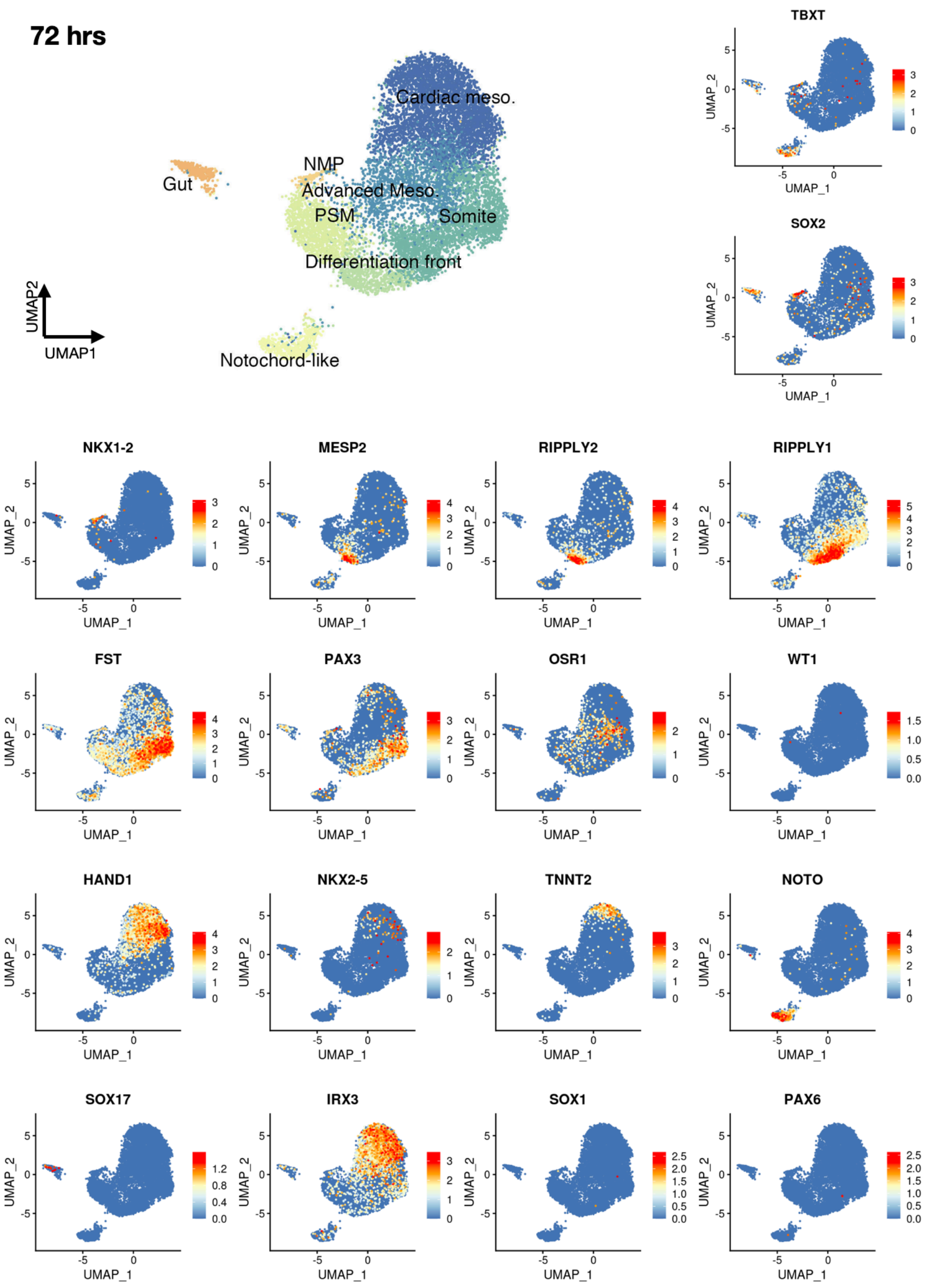

Supplementary Fig.3. Marker gene expression & cell type annotation in human gastruloids at 72 hrs after induction.

UMAP with unsupervised clustering of scRNA-seq profiles is shown, along with expression of marker genes used to annotate these clusters as a continuum of neuromesodermal progenitors, presomitic mesoderm, differentiation front, and somites; additionally, advanced mesoderm and cardiac mesoderm, as well as notochord-like cells and gut/endoderm-like cells, were detected. NMP, neural mesodermal progenitor; PSM, presomitic mesoderm.

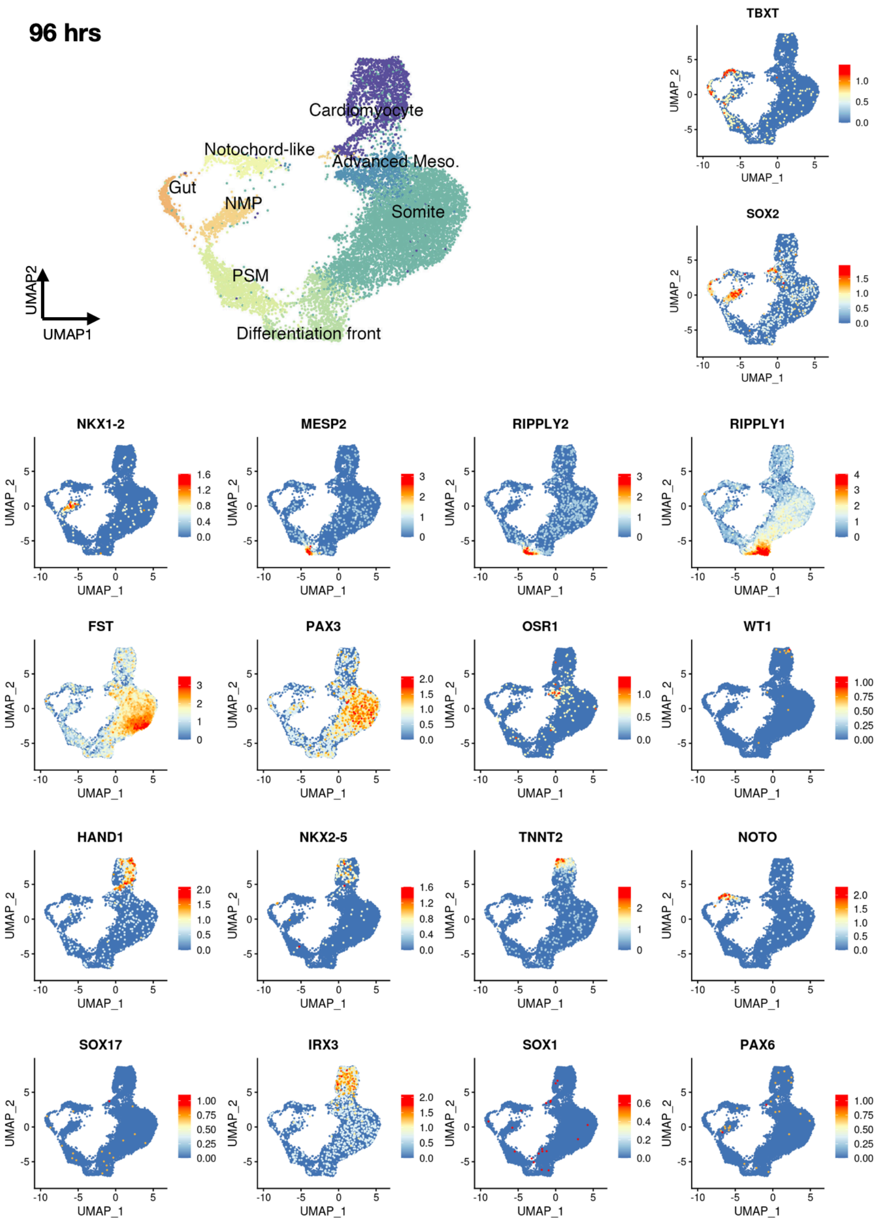

Supplementary Fig.4. Marker gene expression & cell type annotation in human gastruloids at 96 hrs after induction.

UMAP with unsupervised clustering of scRNA-seq profiles is shown, along with expression of marker genes used to annotate these clusters as a continuum of neuromesodermal progenitors, presomitic mesoderm, differentiation front, and somites; additionally, advanced mesoderm and cardiac mesoderm, as well as notochord-like cells and and gut/endoderm-like cells, were detected. NMP, neural mesodermal progenitor; PSM, presomitic mesoderm.

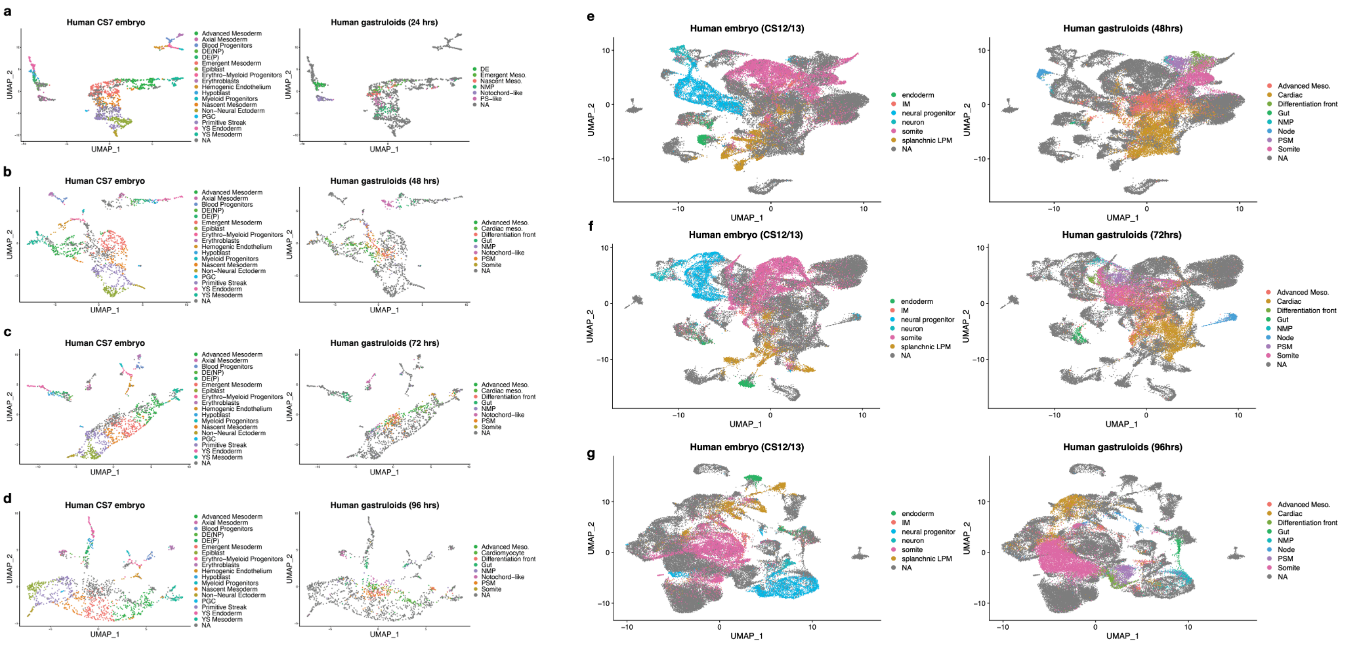

Supplementary Fig.5. Co-embedding of human gastruloid scRNA-seq profiles (24-96 hrs) with published data from either CS7 or CS12/13 human embryos.

Co-embedding and UMAP visualisation of scRNA-seq profiles of human embryos (left) with human gastruloids (right) at CS7 (a-d) or at CS12-13 (e-g)[24]. In each panel, the same UMAP visualisation is shown twice, but with different subsets of cells labelled. NA refers to cells from the other sample in the co-embedding, which are instead annotated in the other column. As noted in the text, integration of scRNA-seq data from 24 hrs with CS7 data was informative with respect to cell type annotation. However, projections of 48-96 hr data with either CS7 or CS12/13 human embryo scRNA-seq profiles were not informative, potentially because the stages represented by these gastruloids fall in between CS7 and CS12/13.

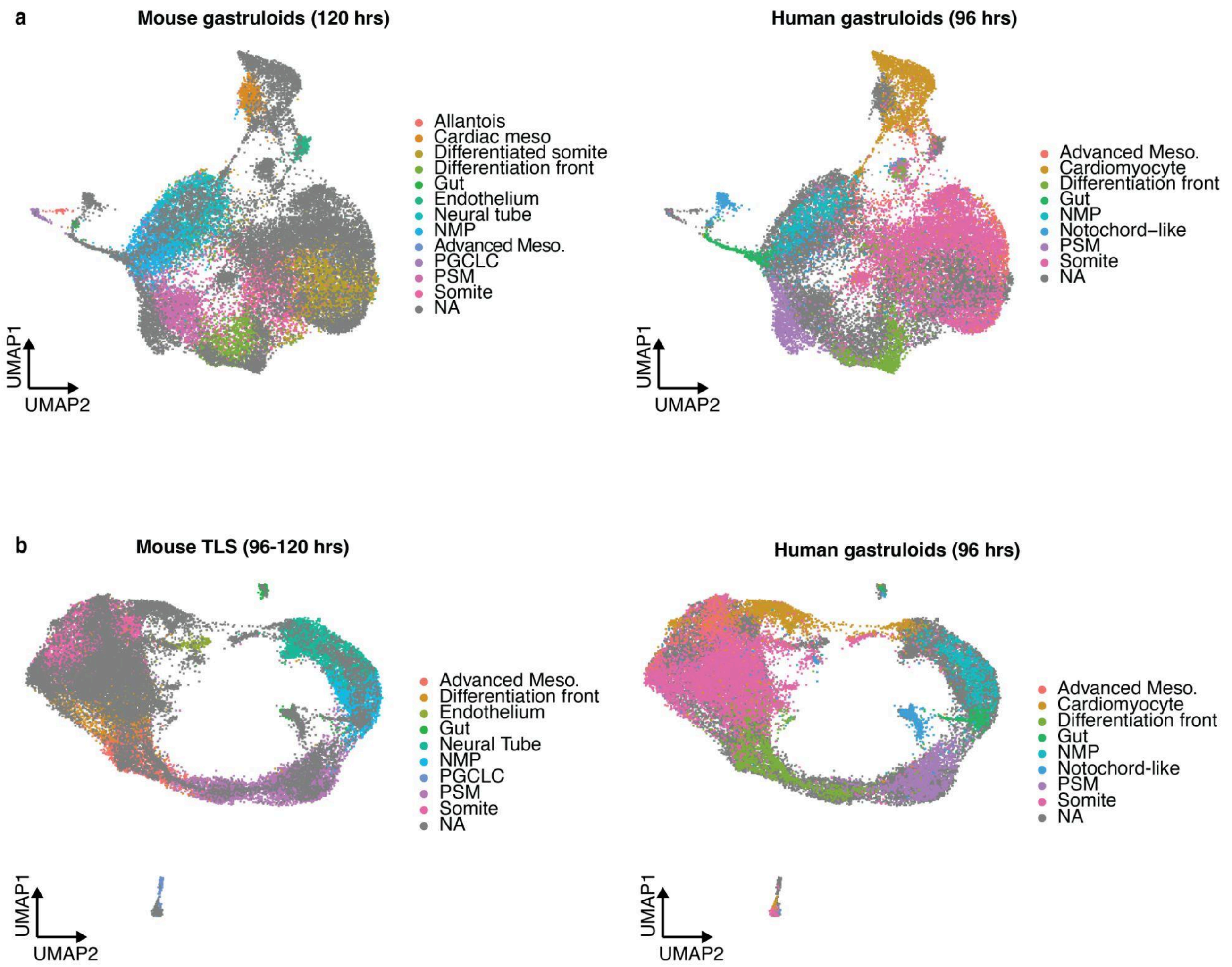

Supplementary Fig.6. Co-embedding of human gastruloid scRNA-seq profiles (96 hrs) with published data from two mouse gastruloid models.

a, Co-embedding and UMAP visualisation of scRNA-seq profiles, labelled by annotations from 120 hrs mouse gastruloids[10] (left) or 96 hrs human gastruloids (this study, right). NA refers to cells from the other sample in the co-embedding, which are instead annotated in the other column. **b**, Co-embedding and UMAP visualisation of scRNA-seq profiles, labelled by annotations from 96-120 hrs mouse TLS[15] (left) or 96 hrs human gastruloids (this study, right).

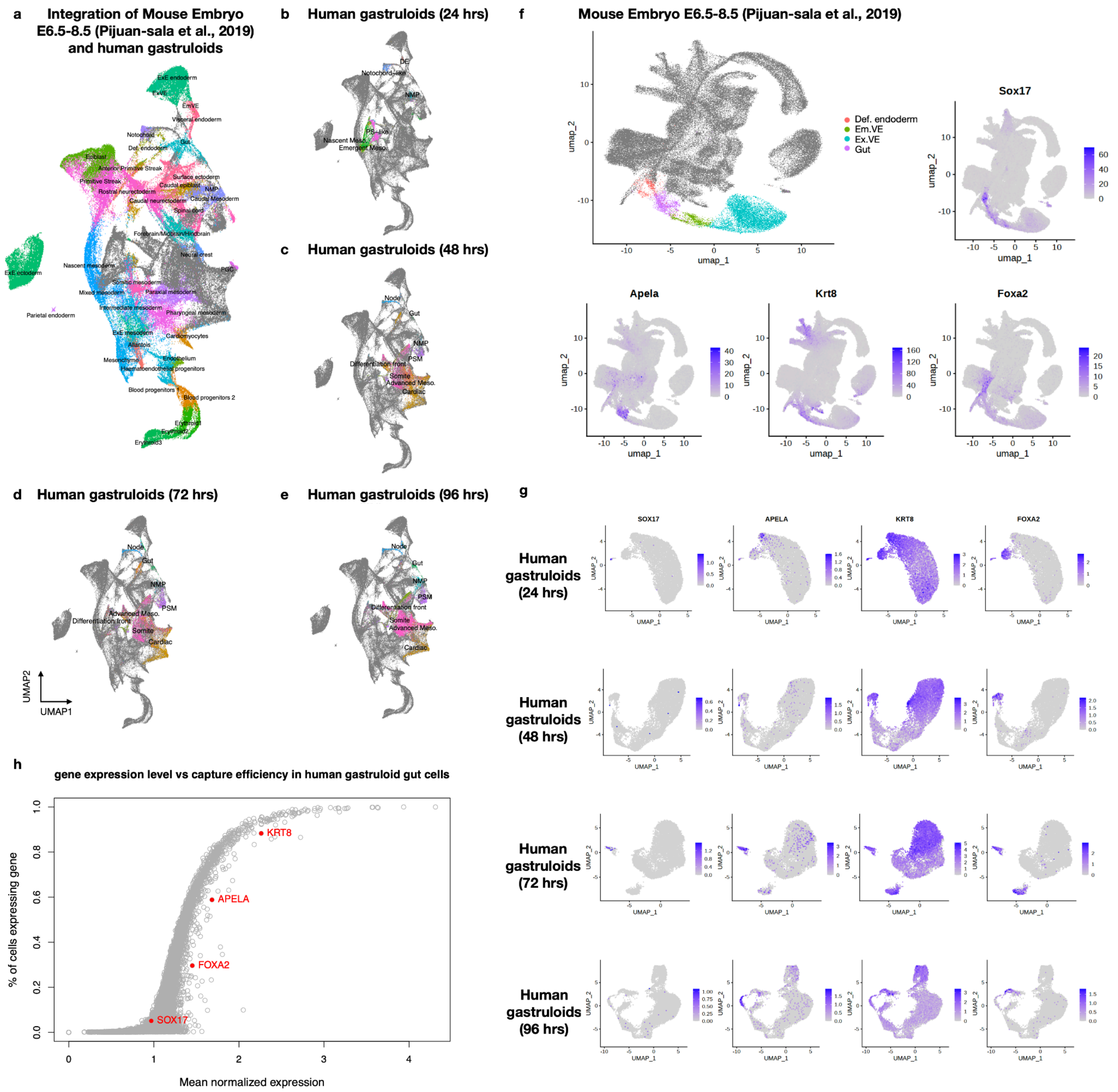

Supplementary Fig.7. Mapping gut/endoderm-like cells via integration of human gastruloid and mouse embryo scRNA-seq data.

a, Co-embedding and UMAP visualisation of scRNA-seq profiles from 24-96 hrs human gastruloids (this study) and E6.5-E8.5 mouse embryos[80]. Mouse embryo cells are coloured and annotated with labels from the original study. **b-e**, Same as panel (**a**), with human gastruloid cells from 24 hrs (**b**), 48 hrs (**c**), 72 hrs (**d**) or 96 hrs (**e**), coloured and annotated with labels from this study. Cells that we have annotated as resembling definitive endoderm (DE) at 24 hrs co-localize with mouse embryonic visceral endoderm (EmVE) cells. Cells that we have annotated as gut/endoderm-like at 48-96 hrs co-localize with mouse embryonic gut cells. **f**, Expression of gut marker genes (*Sox17*, *Apela*, *Krt8*, *Foxa2*) in mouse embryos (E6.5-E8.5) during the transition of definitive endoderm to gut. **g**, Visualisation of the same gut marker genes in the development of human gastruloids. **h**, Variable *SOX17* expression in gut/endoderm-like cell type across timepoints is possibly due to its relatively low detection in 10X Genomics data. For all endoderm/gut cells in human gastruloids (24-96 hrs) profiled with 10x Genomics scRNA-seq, the percentage of cells that expressed each gene was calculated. Among cells that express a specific gene, the average expression level (log-normalised) of that gene was further computed. The diagram shows correlation of the percentage of cells expressing a gene vs. the average expression level for that same gene. The selected gut marker genes (*i.e. KRT8, APELLA, FOXA2, SOX17*) are labelled in red. *SOX17* transcripts are poorly captured in 10X Genomics data, relative to other gut marker genes.

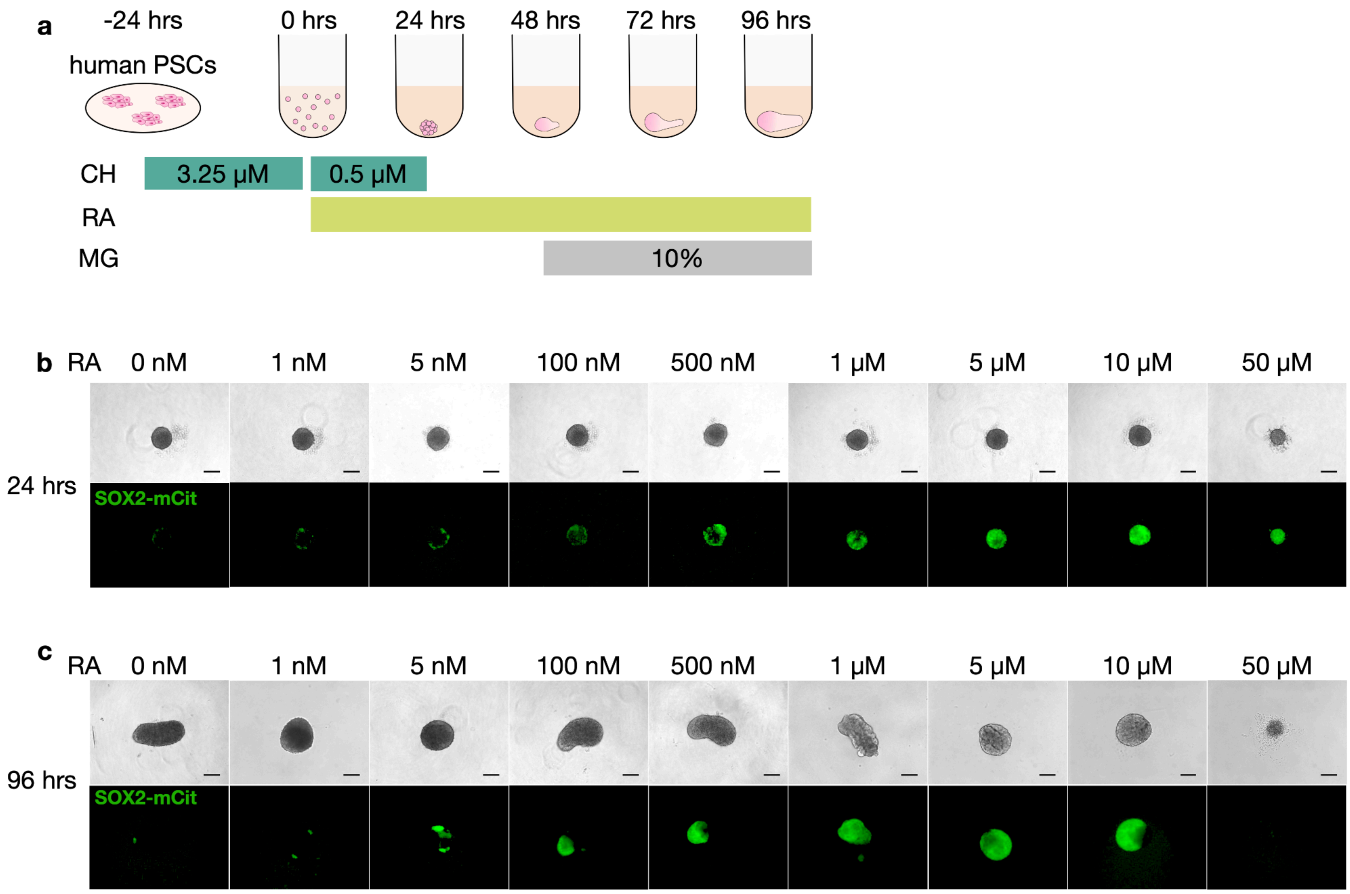

Supplementary Fig.8. Effect of continuous retinoic acid on neural cell induction in human gastruloids.

a, Schematic of RA and Matrigel treatment of human gastruloids. RA, retinoic acid; MG, 10% Matrigel. **b**-**c**, Representative images of RA and Matrigel-treated human gastruloids at 24 hrs (**b**) and 96 hrs (**c**). The intensity of SOX2-mCit correlated with the concentration of RA. Scale bars, 100 μm, N = 48 for each condition.

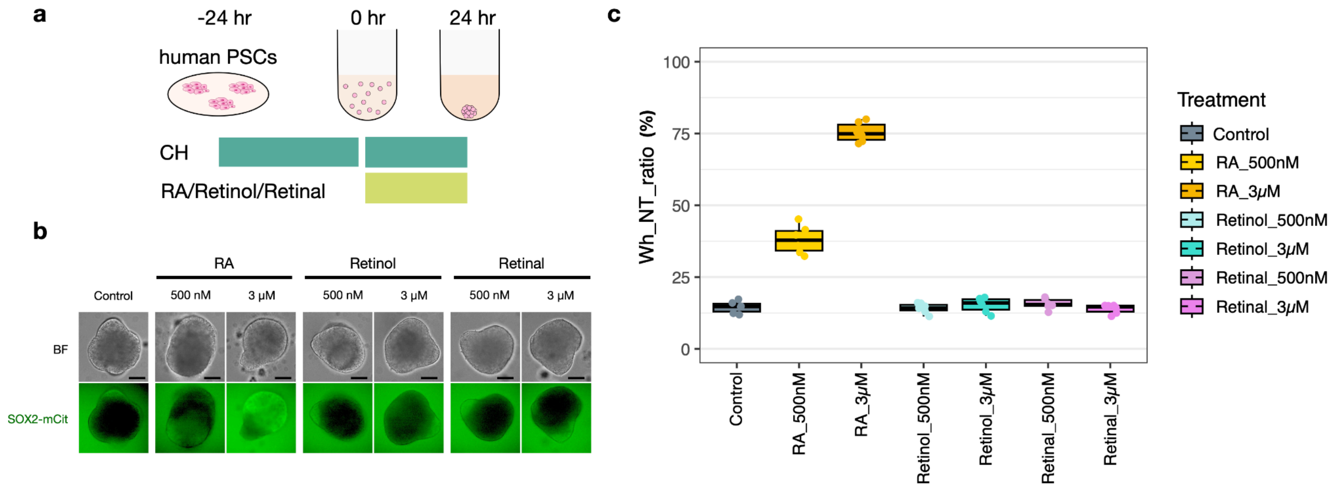

Supplementary Fig.9. The effects of RA/Retinol/Retinal on neural fate induction in human gastruloids.

a, Schematic for the induction of human gastruloids with RA, retinol or retinal supplementation. RA, retinol or retinal were added to the medium at 0-24 hrs. **b**, Brightfield and mCit fluorescent images of 48 hrs human gastruloids after addition of retinol, retinal or RA at 0-24 hrs. In this cell line, mCit fluorescence is indicative of SOX2 expression in human gastruloids. Scale bar, 100 μm. **c**, Quantification of the SOX2-mCit signals. N ≥ 6 gastruloids per condition.

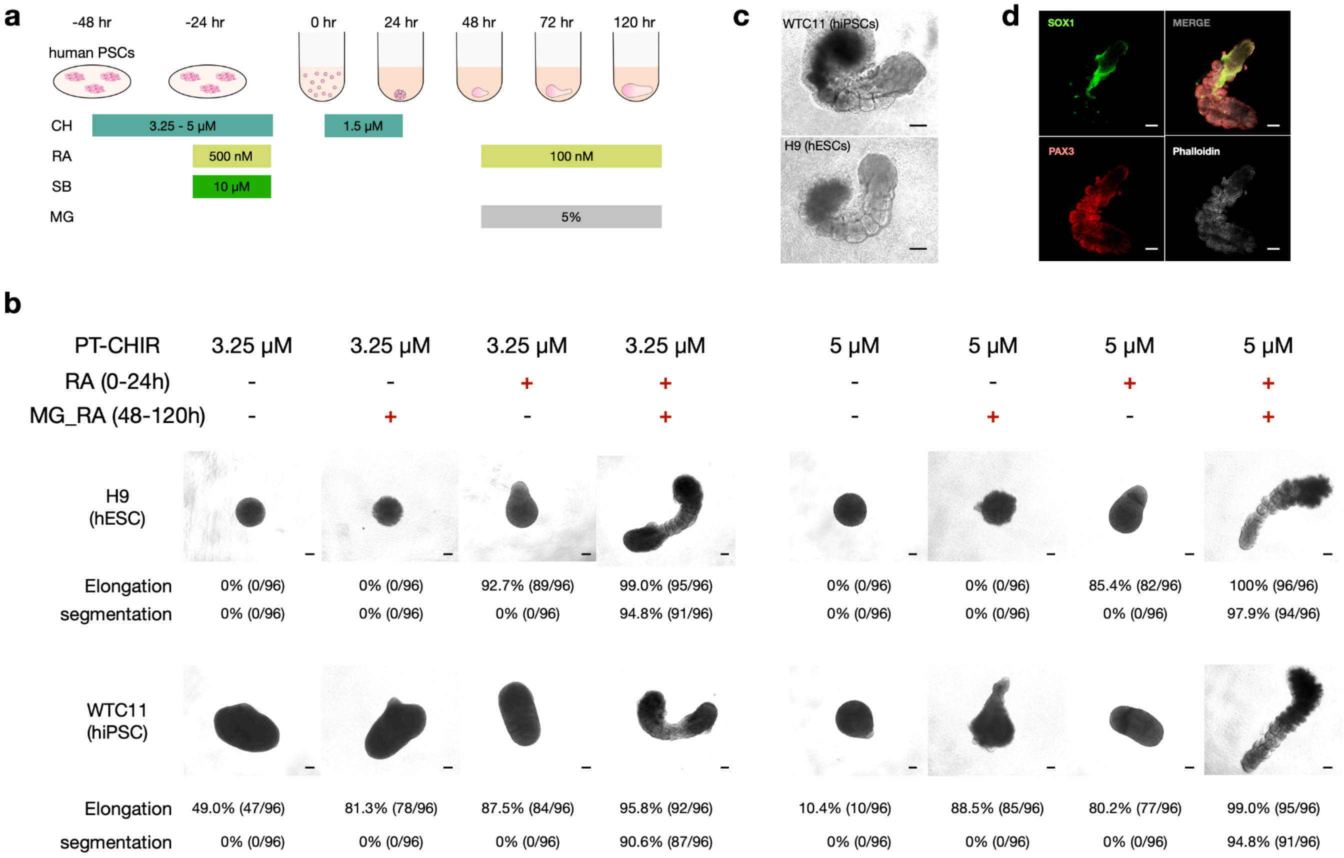

Supplementary Fig.10. RA-gastruloid induction from human iPSCs (WTC11) and human ESCs (H9).

a, Scheme for pre-treatment and human RA-gastruloid induction. RA, retinoic acid; MG, Matrigel; CH; CHIR99021; SB, SB-431542. **b**, Effects of concentration of CHIR at pre-treatment (PT-CH), supplemented with RA at 0-24 hrs and RA and 5% Matrigel at 48-120 hrs. Scale bar, 100 µm. **c**, Representative images of RA-gastruloids derived from human iPSCs (WTC11, top) and another human ESC line (H9). **d**, Immunostaining of WTC11-derived RA-gastruloids with SOX1-, PAX3-antibodies and Phalloidin. Scale bar, 100 µm. N = 14 out of 17 RA-gastruloids showed similar marker gene expression patterns,

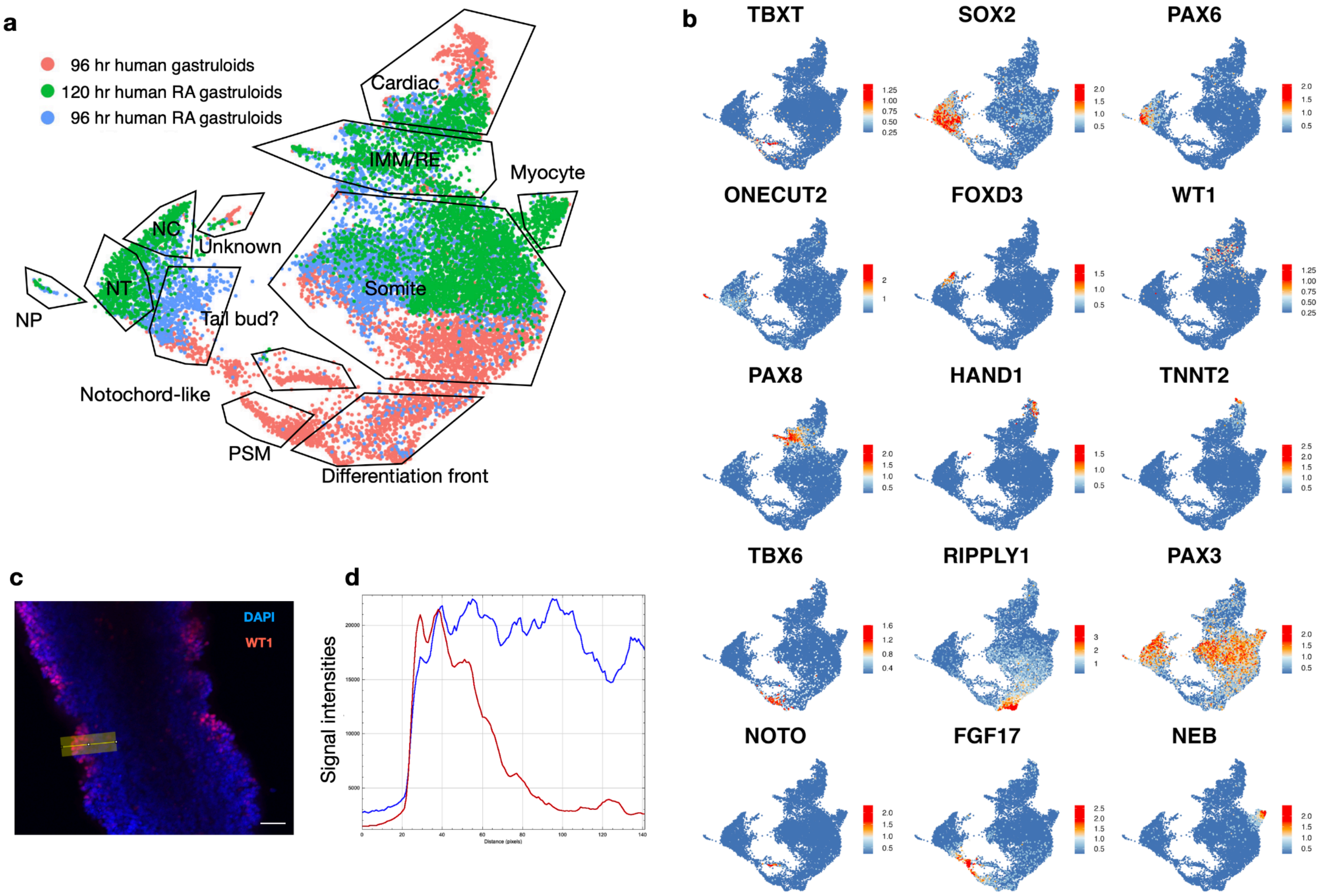

Supplementary Fig.11. Comparison of cell types detected in human conventional vs. RA-gastruloids.

a, Co-embedded UMAP of scRNA-seq data coloured by the sample of origin. Red: 96 hrs conventional gastruloid (N = 5,000 cells). Blue: 96 hrs RA-gastruloid (N = 5,000 cells). Green: 120 hrs RA-gastruloid (N = 5,000). Clusters were annotated based on marker gene expression patterns. NP, neural progenitors; NC, neural crest; NT, neural tube; PSM, presomitic mesoderm; IMM, intermediate mesoderm; RE, renal epithelium; NMP, neuromesodermal progenitor. **b**, Marker gene expression patterns in co-embedded UMAP. **c**, Immunostaining of WT1 in 120 hrs RA-gastruloids. Signal intensities of DAPI (blue) and WT1 (red) were measured on the area indicated by the yellow line. Scale bar, 100 μm. **d**, Measured intensities of DAPI (blue) and WT1 (red) signals. Although there may be lower DAPI staining at more internal positions, a very sharp dropoff in WT1 staining is observed at depths where DAPI signal is entirely maintained.

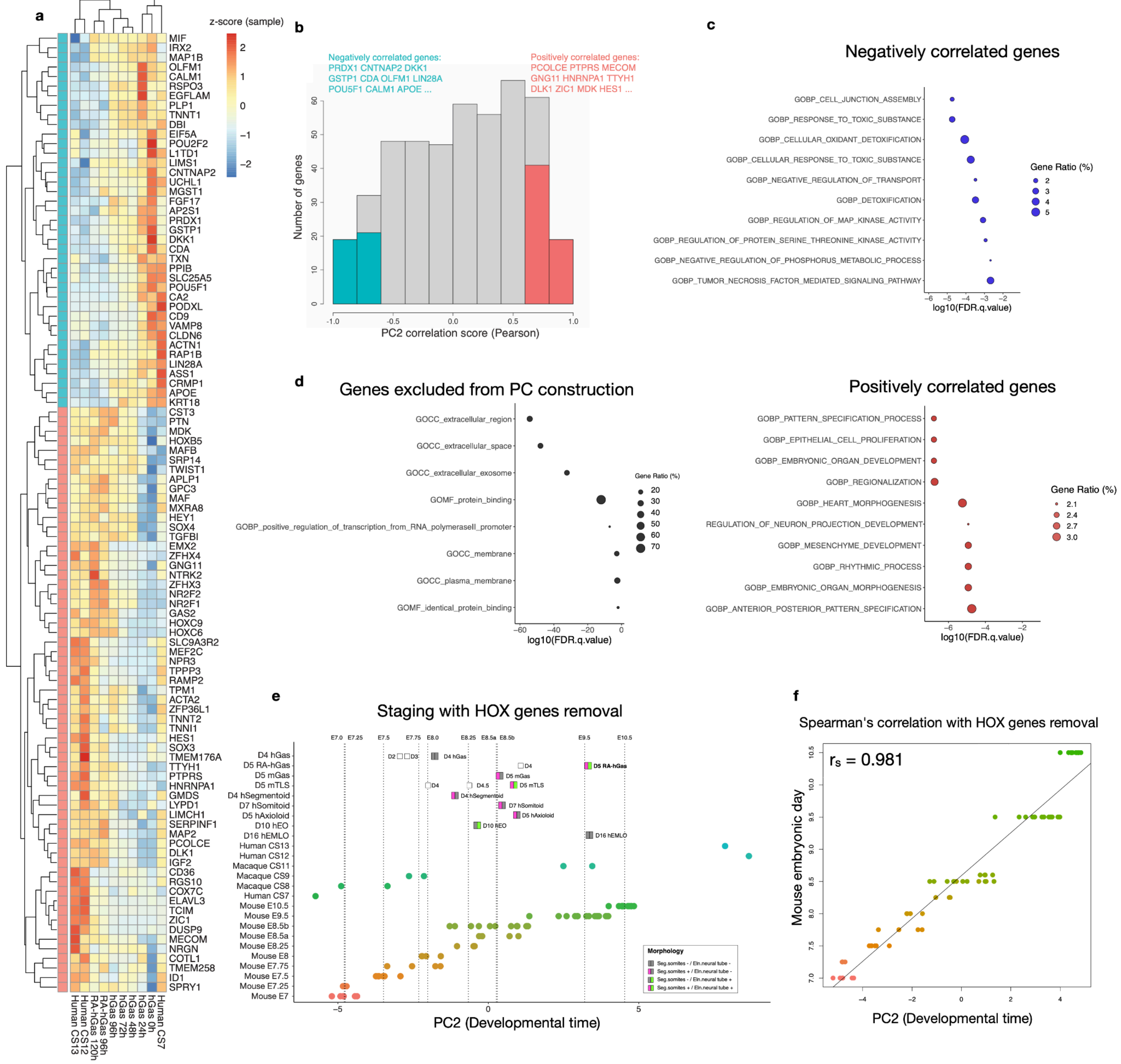

Supplementary Fig.12. Genes and pathways that are correlated with developmental progression.

a, Biclustered heatmap shows the z-transformed expression levels of each of these top 100 genes (rows) positively (pink in column at left) or negatively (blue in column at left) correlated with PC2 in various human samples (columns). Colours correspond to z-scores. **b**, The top 100 genes with the highest absolute correlation scores with PC2 were selected from all 447 genes used for PCA of human samples[24,57]. **c**, Gene Ontology (GO) enrichment analysis (MSigDB v7.5.1) of the genes negatively (Top) or positively (Bottom) correlated with PC2[83,84]. Gene Ratio: percentage of query genes in the matched GSEA gene sets. **d**, GO term enrichment computed by DAVID Bioinformatics Resources (https://david.ncifcrf.gov/) on all the human embryo variable genes excluded from the final PCA analysis. **e**, Similar to **Fig. 4e**, but after removing HOX genes. **f**, Scatterplot and Spearman's correlation of mouse embryos' PC2 values (x-axis) and their embryonic stage (y-axis; E7.0-10.5), after removing HOX genes. A fitted regression line from a linear model is plotted. r_s, Spearman's correlation.

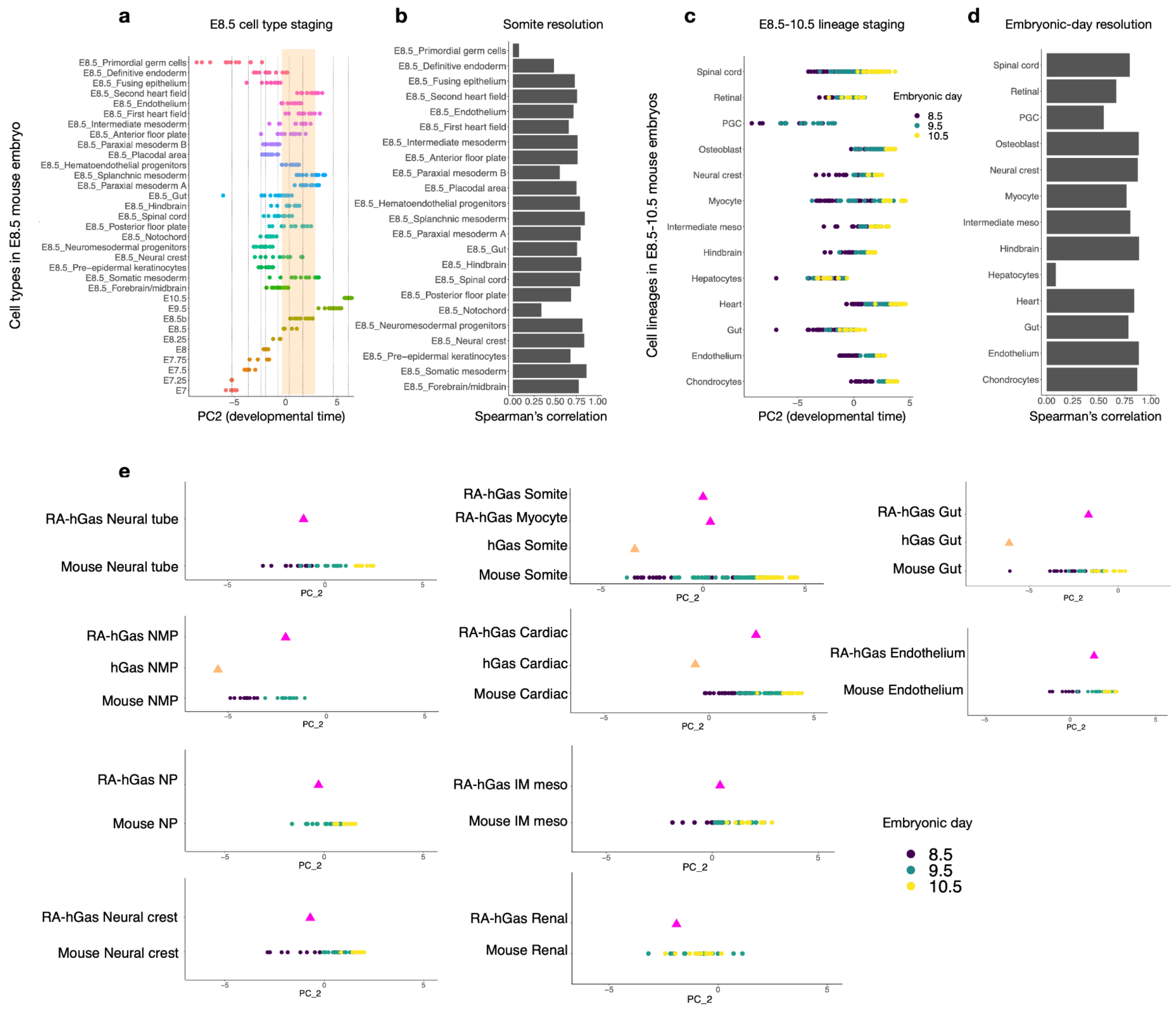

Supplementary Fig.13. Developmental staging of individual cell types within human RA-gastruloids against *in vivo* mouse development.

a, Major cell types from E8.5 mouse embryos[58,59] (y-axis) were mapped onto the human-derived PC2 axis (x-axis). For top rows, each datapoint represents a pseudo-bulk transcriptome of the cell type in an individual embryo (E8.5b data generated by sci-RNA-seq3), while bottom rows correspond to pseudobulk embryos (E8.5b-E10.5) or embryo pools (E7-E8.5). Orange shaded area corresponds to PC2 range spanned by E8.5 pseudobulk embryos. **b,** Spearman's correlation of the PC2 values of individual mouse cell types vs. somite count. **c,** Major cell types from E8.5-E10.5 mouse embryos[58,59] (y-axis) were mapped onto the human-derived PC2 axis (x-axis). Although they are not fully aligned to one another, within any given cell type, we observe that the PC2 values of E9.5 mice are generally higher than E8.5 mice, and E10.5 mice generally higher than E9.5 mice. **d,** Spearman's correlation of the PC2 values of individual mouse cell types vs. embryonic day (E8.5-E10.5). **e,** For various cell types observed in 120 hrs human RA-gastruloids, we plot the PC2 values of either the pseudobulked cell type from pooled human RA-gastruloids (top rows) or individual mouse embryos (E8.5-E10.5, bottom rows). NMP, neural-mesodermal progenitor; NP, neural progenitor; IM meso, intermediate mesoderm.

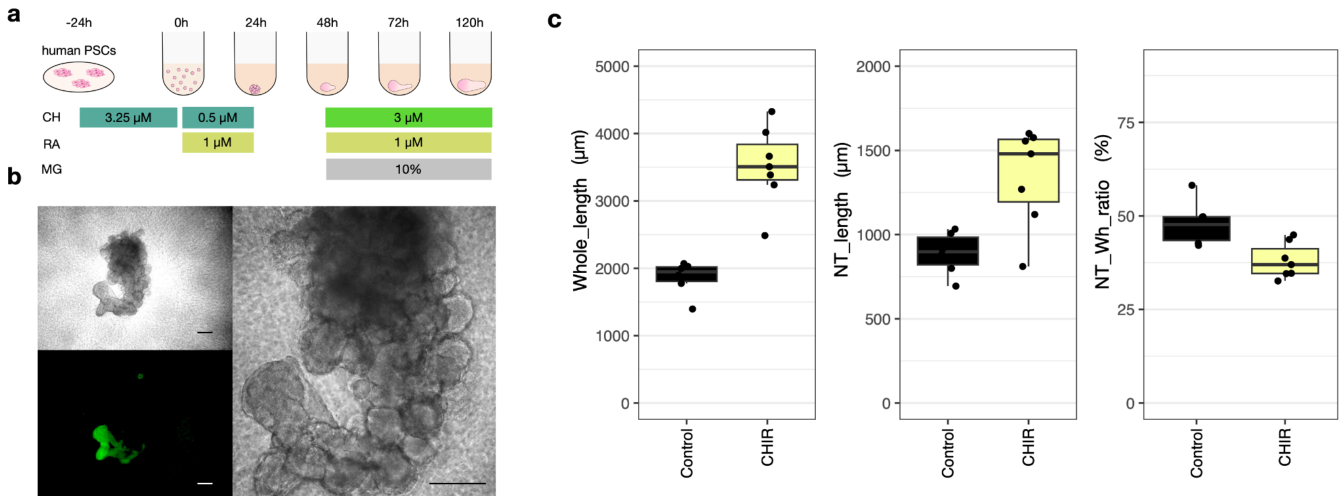

Supplementary Fig.14. Hyperactivation of WNT signalling in human RA-gastruloids.

a, Schematic of experimental hyperactivation of WNT signalling in human RA-gastruloids. In brief, the protocol was modified to add CHIR back into the system at 48 hrs. CHIR, CHIR99021; MG, Matrigel; RA, Retinoic acid. **b**, Representative morphology of CHIR-treated 120 hrs RA-gastruloids. Bar, 100 µm. N =192 human RA-gastruloids generated under the modified protocol showed similar morphology (elongated gastruloid with a high density of somite-like structures appearing along the entire anterior-posterior axis) (Top left) Bright field, (bottom left) SOX2-mCit, and (right) magnified view of top left panel. Scale bar, 100 µm. **c**, Morphometrics as described in **Fig. S14.** (from left to right) Full length (µm), neural tube length (µm) measured with SOX2-mCit signal, and neural-tube/full-length ratio of 120 hrs human RA-gastruloids. N = 6 and 7 for 120 hrs controls and CHIR treatment, respectively.

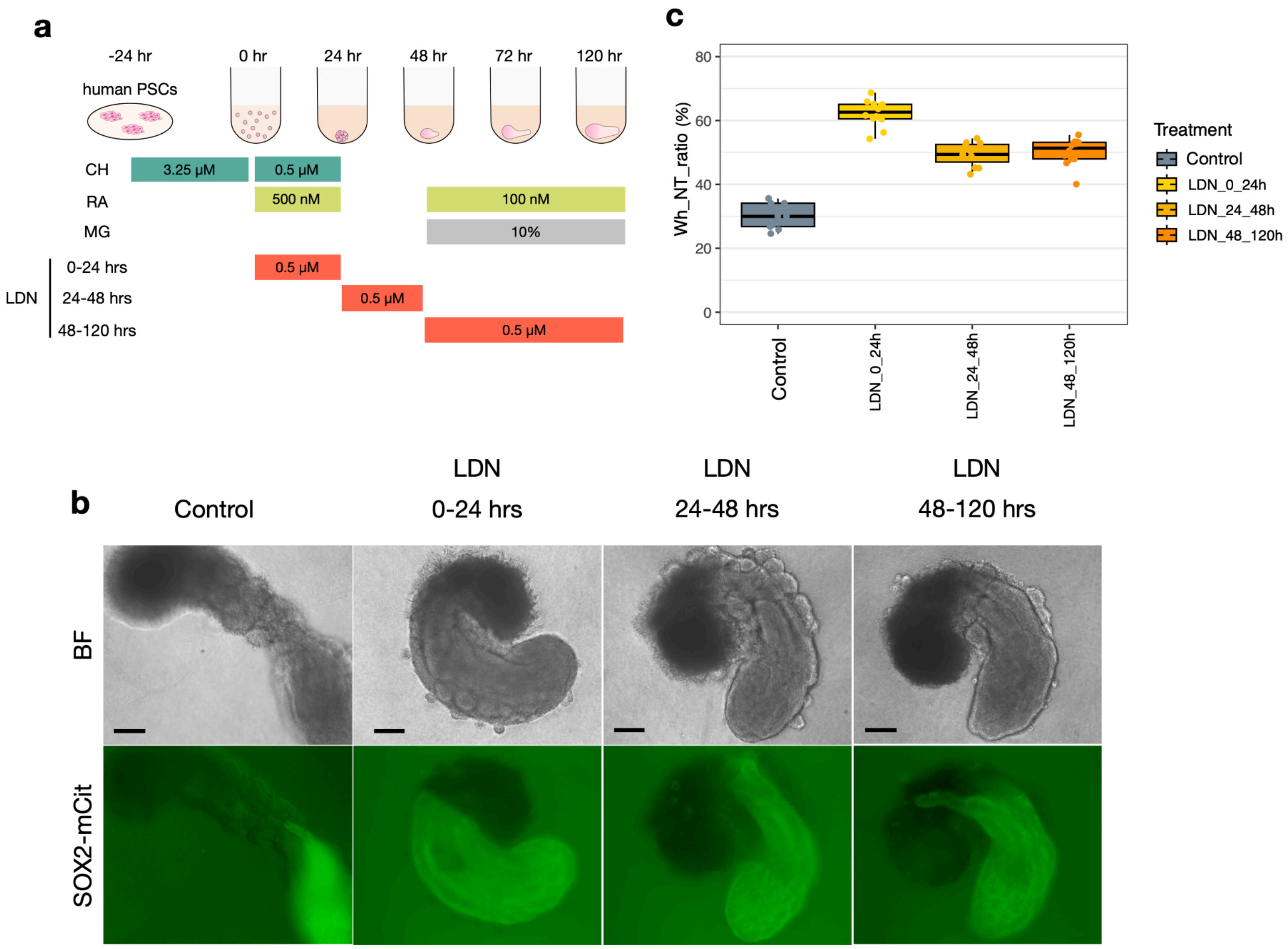

Supplementary Fig.15. Temporal inhibition of BMP signalling.

a, Schematic of temporal inhibition of BMP signalling in human RA-gastruloids. RA, retinoic acid; MG, Matrigel; CH; CHIR, CHIR99021; LDN, LDN183189. **b**, Representative bright-field (top) or SOX2-mCit (bottom) images of 120 hrs human RA-gastruloids with no LDN treatment (control) vs. treatment at 0-24 hrs, 24-48 hrs or 48-120 hrs. Scale bar, 100 µm. **c**, Quantification of SOX2-mCit area in human RA-gastruloids upon LDN treatment. N = 9, 12, 11, 12 for controls or 0-24 hr, 24-48 hr, 48-120 hrs LDN treatment, respectively.

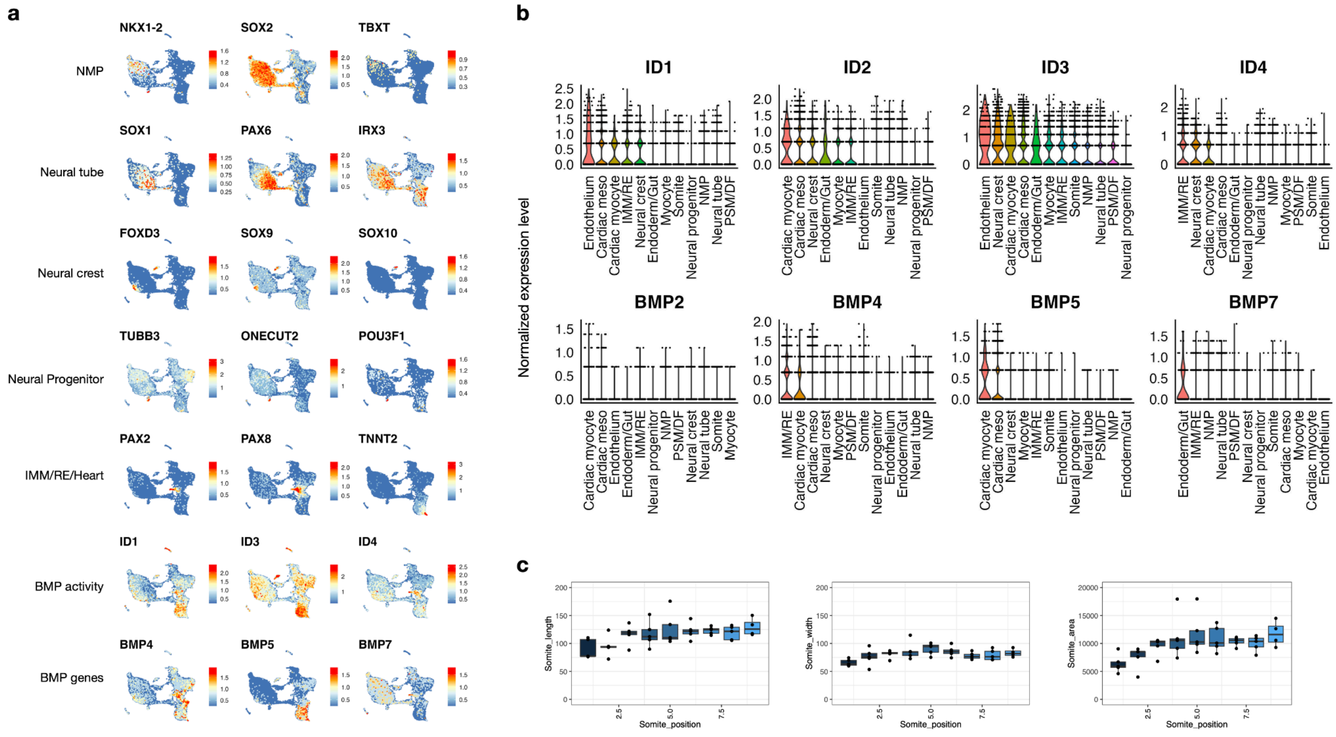

Supplementary Fig.16. Effects of LDN treatment on gene expression and somite morphology in human RA-gastruloids.

a, UMAP visualisation of co-embedded scRNA-seq data from untreated and LDN-treated human RA-gastruloids. The same projection is shown in **Fig. 5d**. Gene expression of three marker genes for each cell type are shown in each row, with two additional rows for markers of BMP activity (second to last row) or BMP genes (last row). **b**, Violin plots showing ID and BMP gene expression patterns for various cell types. Cell types along x-axes are sorted by the mean expression levels of the gene, in descending order. **c**, Quantification of somites in LDN-treated RA-gastruloids. Length, width and area of somites in LDN-treated RA-gastruloids as a function of position. N = 5 gastruloids.

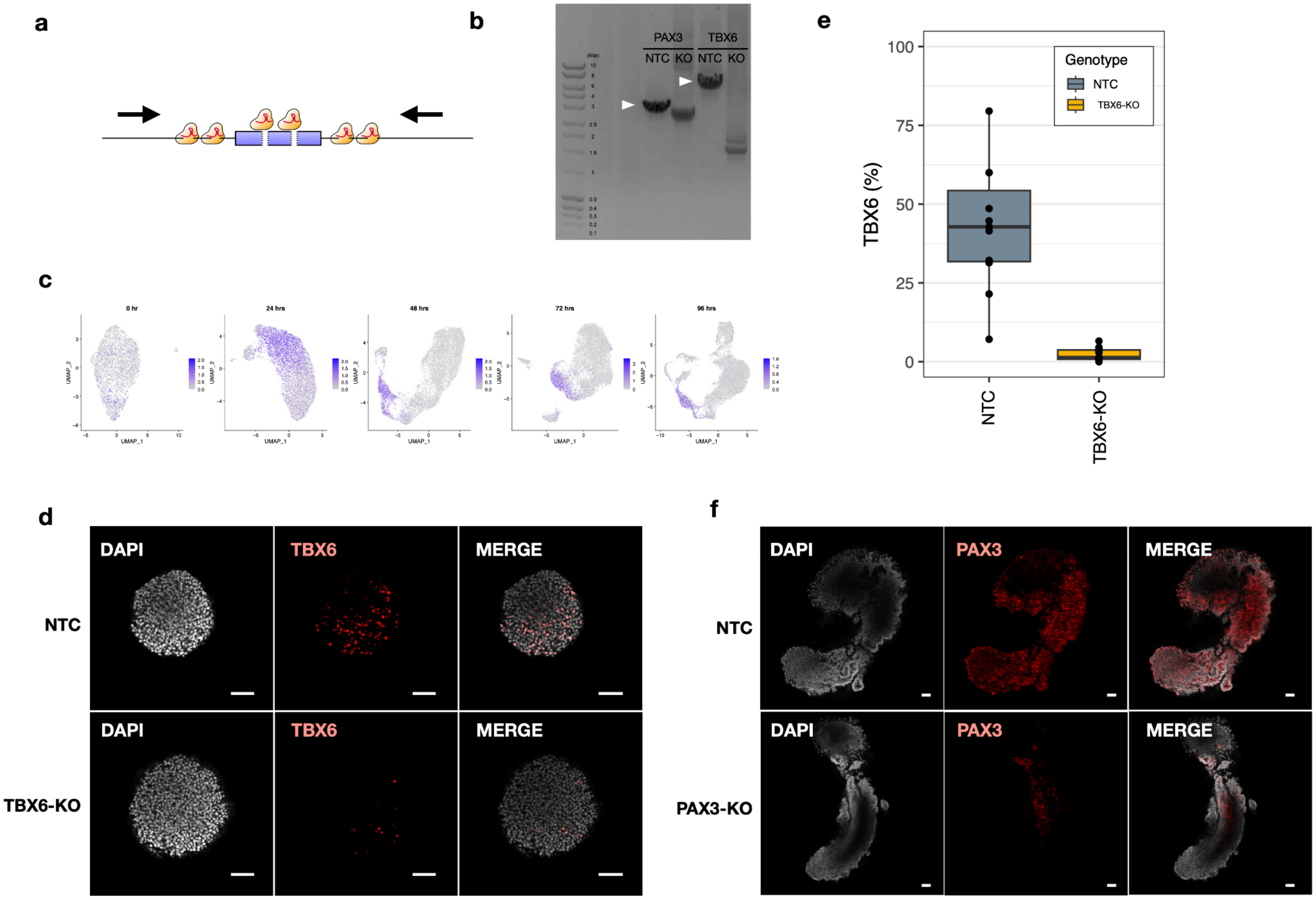

Supplementary Fig.17. Validation of knockouts in TBX6-KO or PAX3-KO human RA-gastruloids.

a, Schematic for genotyping PCR. Forward and reverse primers are indicated as arrows. Primers were set outside of the multiple Cas9-targeting sites. **b**, Genotyping PCR on wildtype and Cas9-RNP induced hPSCs. Transfected hPSCs were harvested and lysed at day 7 from the transfection. The predicted wildtype band sizes are shown in arrowheads. **c**, *TBX6* expression patterns in scRNA-seq data from conventional human gastruloids, 0-96 hrs. **d**, Representative images of immunostaining of TBX6 protein in NTC control and TBX6-KO RA-gastruloids at 24 hrs. Scale bar, 100 µm. **e**, Estimated proportion of TBX6+ cells in NTC controls or TBX6-KO RA-gastruloids. For panels (**d-e**), N = 11 and 14 for control and TBX6-KO gastruloids, respectively. **f,** Representative images of immunostaining of PAX3 protein in NTC control and PAX3-KO RA-gastruloids at 120 hrs. Scale bar, 100 µm. N = 5 and 6 for control and PAX3-KO gastruloids, respectively.

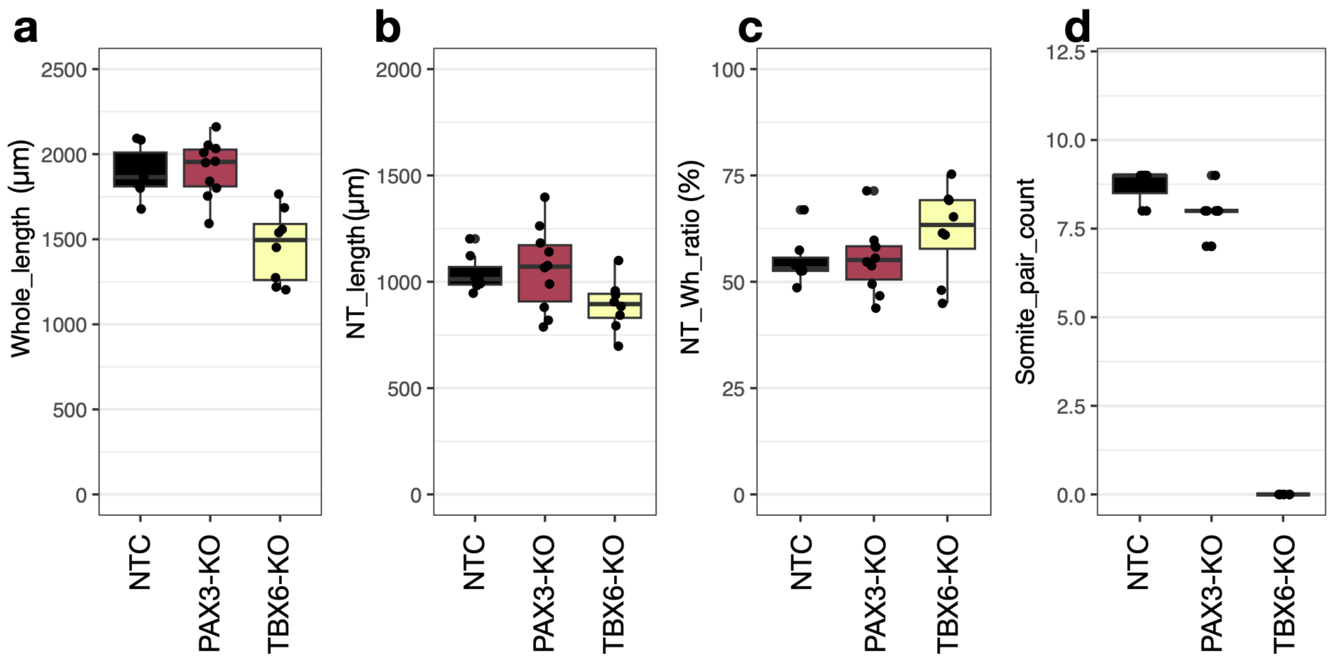

Supplementary Fig.18. Morphometrics of PAX3-KO and TBX6-KO human RA-gastruloids.

Morphological characteristics were measured as in **Fig. S14.** **a**, Full length (μm) of 120 hrs human RA-gastruloids, stratified by genotype. **b**, Neural tube length (μm) measured with SOX2-mCit signals, stratified by genotype. **c**, Neural-tube/full-length ratio, stratified by genotype. **d**, Somite pair counts, stratified by genotype. N = 7, 10, and 8 for non-targeting control (NTC), PAX3-KO, and TBX6-KO RA-gastruloids, respectively.

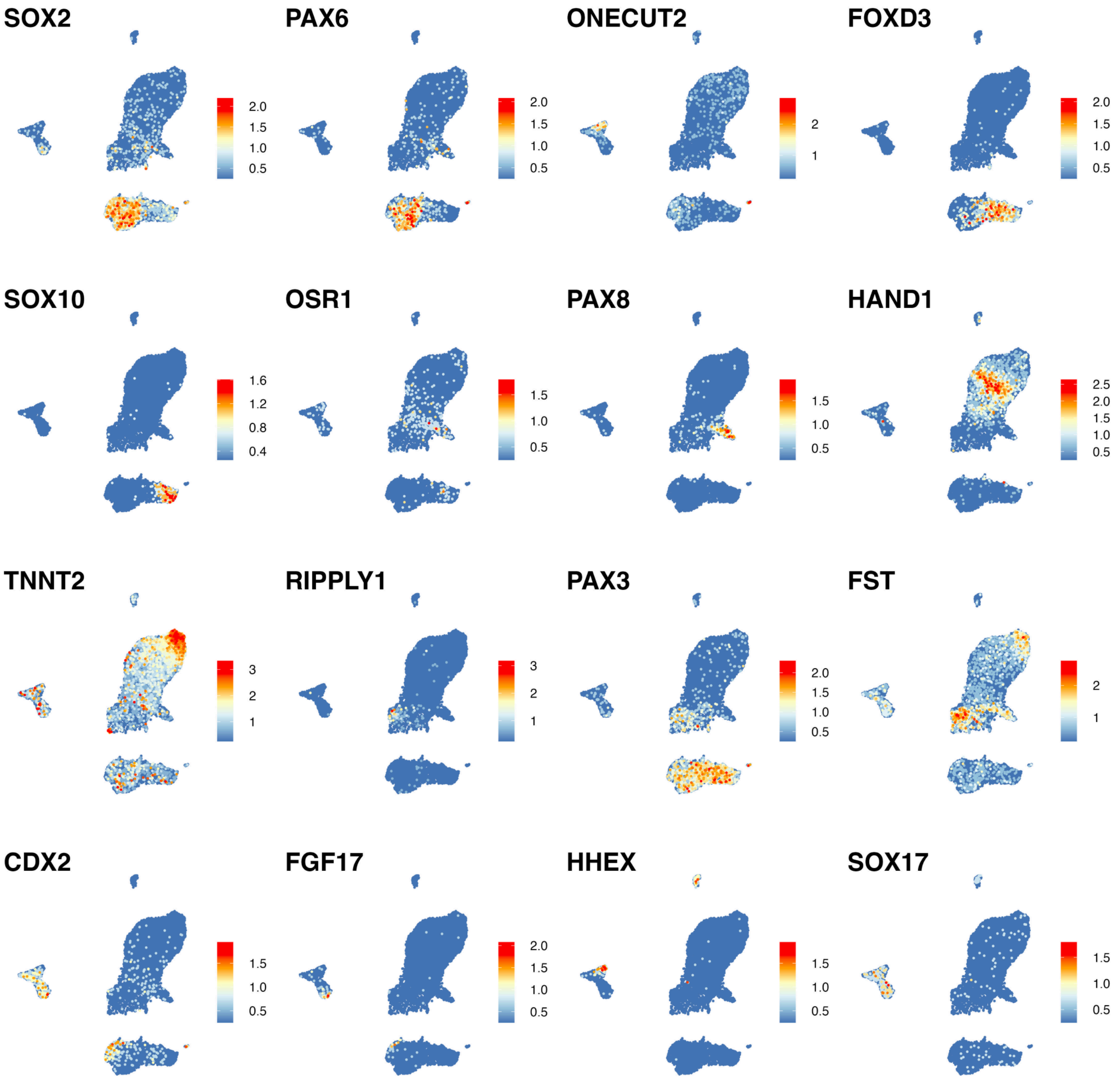

Supplementary Fig.19. Marker gene expression in NTC and genetically-perturbed human RA-gastruloids

Marker gene expression in NTC and genetically-perturbed human RA-gastruloids on the co-embedded UMAP also shown in **Fig. 6d**. Marker genes are for the following cell types: *SOX2*, neural cells; *PAX6*, neural tube; *ONECUT2*, neural progenitor; *FOXD3* and *SOX10*, neural crest; *OSR1*, intermediate mesoderm; *PAX8*, renal epithelium; *HAND1*, cardiac mesoderm; *TNNT2*, cardiac myocyte; *RIPPLY1*, differentiation front; *PAX3* and *FST*, somite; CDX2, caudal cell; *FGF17*, definitive endoderm; *HHEX*, gut; *SOX17*, pan-endoderm.

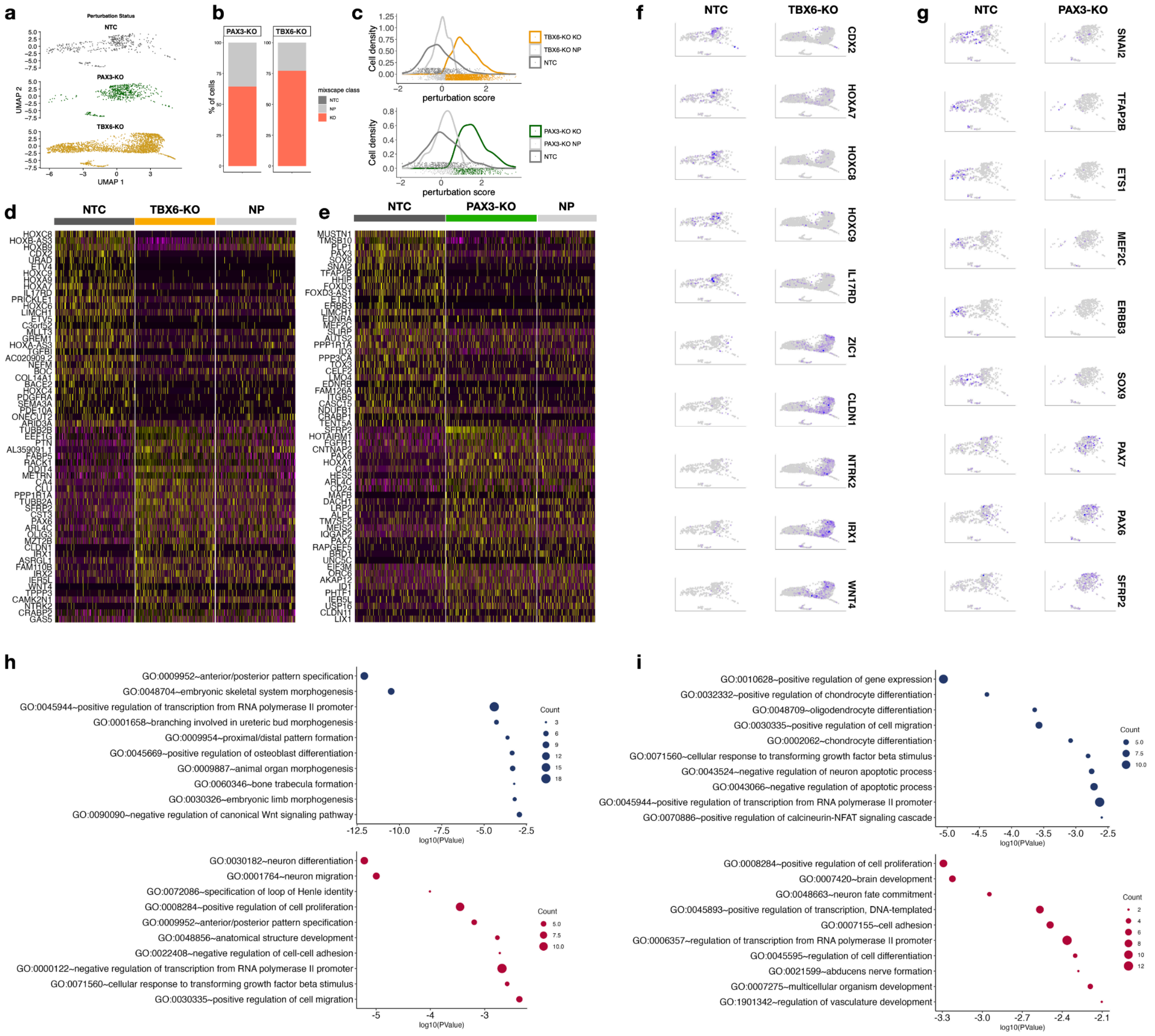

Supplementary Fig.20. Effects of genetic perturbation on human RA-gastruloids.

a, UMAP visualisation of neural cell types (NMP, neural tube, neural crest, and neural progenitor) of NTC, TBX6-KO, and PAX3-KO human RA-gastruloids. **b**, Bar plots representing the results of Mixscape classification of TBX6-KO and PAX3-KO RA-gastruloids. Cells were classified as knocked out (KO) or non-perturbed (NP), based on the perturbation scores calculated by Mixscape. **c**, Distribution of Mixscape perturbation scores. **d-e**, single cell RNA heatmaps of DEGs in TBX6-KO and PAX3-KO RA-gastruloids. The top DEGs (30 up, 30 down) for TBX6-KO (**d**) and PAX3-KO (**e**) RA-gastruloids are shown. **f-g**, Expression patterns of representative DEGs of TBX6-KO (**f**) and PAX3-KO (**g**) projected onto the same UMAP shown in panel (**a**). **h-i**, GO enrichment analysis on (Top) down-regulated and (Bottom) up-regulated DEGs in TBX6-KO (**h**) and PAX3-KO RA-gastruloids (**i**).

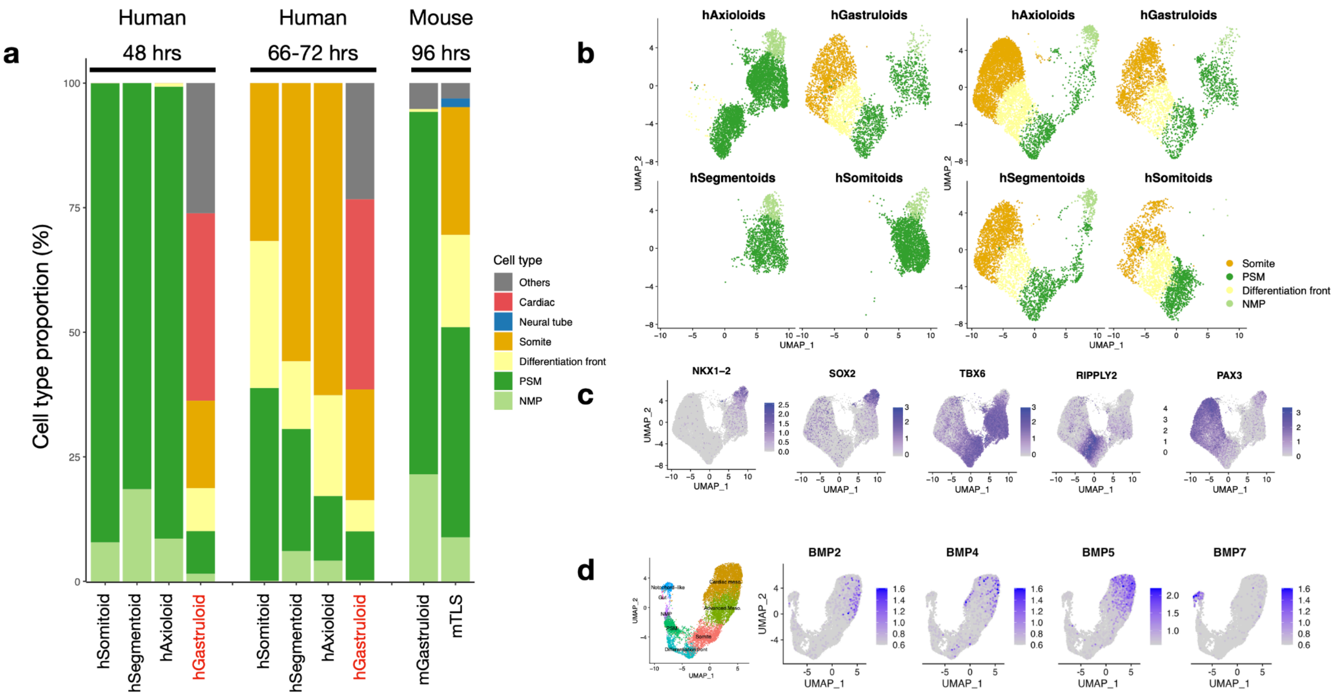

Supplementary Fig.21. Cell type composition of embryo models.

a, Barplots showing the percentage of each cell type in various embryo models at 48 to 72 hrs (human) or 96 hrs (mouse). "Others" includes advanced mesoderm, node and gut for hGastruloids; ectopic pluripotency and gut for mGastruloids; and PCGLC, endothelial, and gut for mTLS. Models shown include conventional human gastruloids (hGastruloid, 48 and 72 hrs)[11], human somitoids (hSomitoid, 48 and 66 hrs)[16], human segmentoids (hSegmentoid, 48 and 72 hrs)[17], human axioloids (hAxioloid, 48 and 72 hrs)[18], mouse gastruloids (mGastruloid, 96 hrs)[82] and mouse TLS (mTLS, 96 hrs)[15]. **b,** Co-embedding of scRNA-seq data from several human embryo models (axioloids, gastruloids, segmentoids, somitoids) at 48 hrs (left panel) or 72 hrs (right panel), with cells from specific models highlighted in each subpanel, coloured by cell type. **c,** Same co-embedded UMAPs as panel (**b**) with gene expression for markers of NMP (*NKX1-2*, *SOX2*), PSM (*TBX6*), differentiation front (*RIPPLY2*), and somite (*PAX3*) cell types. **d,** UMAP of 48 hrs conventional human gastruloids (left-most panel, same as **Fig. S3**) highlighting gene expression of various BMP transcripts (remaining panels).
