# Supplementary material for: Retinoic acid induces human gastruloids with posterior embryo-like structures
Source: Nat Cell Biol. 2024 Aug 20;26(10):1790–803. doi: 10.1038/s41556-024-01487-8 (PMC11469962; doi:10.1038/s41556-024-01487-8)
Supplement: Supplementary file 2 — Reporting Summary [file 41556_2024_1487_MOESM2_ESM.pdf]

nature portfolio

Corresponding author(s): Nobuhiko Hamazaki
Jay A Shendure

Last updated by author(s): May 7, 2024

Reporting Summary

Nature Portfolio wishes to improve the reproducibility of the work that we publish. This form provides structure for consistency and transparency in reporting. For further information on Nature Portfolio policies, see our Editorial Policies and the Editorial Policy Checklist.

Statistics

For all statistical analyses, confirm that the following items are present in the figure legend, table legend, main text, or Methods section.

n/a	Confirmed	
☐	☒	The exact sample size (*n*) for each experimental group/condition, given as a discrete number and unit of measurement
☐	☒	A statement on whether measurements were taken from distinct samples or whether the same sample was measured repeatedly
☐	☒	The statistical test(s) used AND whether they are one- or two-sided<br>*Only common tests should be described solely by name; describe more complex techniques in the Methods section.*
☐	☐	A description of all covariates tested
☐	☒	A description of any assumptions or corrections, such as tests of normality and adjustment for multiple comparisons
☐	☒	A full description of the statistical parameters including central tendency (e.g. means) or other basic estimates (e.g. regression coefficient) AND variation (e.g. standard deviation) or associated estimates of uncertainty (e.g. confidence intervals)
☐	☒	For null hypothesis testing, the test statistic (e.g. *F*, *t*, *r*) with confidence intervals, effect sizes, degrees of freedom and *P* value noted<br>*Give P values as exact values whenever suitable.*
☒	☐	For Bayesian analysis, information on the choice of priors and Markov chain Monte Carlo settings
☒	☐	For hierarchical and complex designs, identification of the appropriate level for tests and full reporting of outcomes
☐	☒	Estimates of effect sizes (e.g. Cohen's *d*, Pearson's *r*), indicating how they were calculated

Our web collection on statistics for biologists contains articles on many of the points above.

Software and code

Policy information about availability of computer code

Data collection

Base calls were converted to fastq format using cellranger v6.0.0 mkfastq function. The sequencing reads are then demultiplexed based on i5 and i7 barcodes, mapped to hg38 reference genome and assigned to GRCh38 (GENCODE v32/Ensembl 98) genes by cellranger v6.0.0 count function with default setting.

Data analysis

Custom codes were available on https://github.com/shendurelab/Human-RA-Gastruloid.git.
The following common, freely available data analysis software packages were used in this project:
cellranger v6.0.0 (https://support.10xgenomics.com/single-cell-gene-expression/software/pipelines/6.1/release-notes)
Scrublet v0.2.3 (https://github.com/swolock/scrublet.git)
monocle 2.4.0 (https://github.com/cole-trapnell-lab/monocle-release)
data.table_1.15.4 (https://cran.r-project.org/web/packages/data.table/index.html)
harmony_1.2.0 (https://cran.r-project.org/web/packages/harmony/index.html)
Rcpp_1.0.12 (https://cran.r-project.org/web/packages/Rcpp/index.html)
reticulate_1.37.0 (https://cran.r-project.org/web/packages/reticulate/index.html)
lubridate_1.9.3 (https://cran.r-project.org/web/packages/lubridate/index.html)
forcats_1.0.0 (https://cran.r-project.org/web/packages/forcats/index.html)
stringr_1.5.1 (https://cran.r-project.org/web/packages/stringr/index.html)
purrr_1.0.2 (https://cran.r-project.org/web/packages/purrr/index.html)
readr_2.1.5 (https://cran.r-project.org/web/packages/readr/index.html)
tidyr_1.3.1 (https://cran.r-project.org/web/packages/tidyr/index.html)
tibble_3.2.1 (https://cran.r-project.org/web/packages/tibble/index.html)
tidyverse_2.0.0 (https://cran.r-project.org/web/packages/tidyverse/index.html)

dplyr_1.1.4 (https://cran.r-project.org/web/packages/dplyr/index.html)
SeuratObject_4.1.4 (https://rdocumentation.org/packages/SeuratObject/versions/4.1.4)
Seurat_4.4.0 (https://github.com/satijalab/seurat/releases/tag/v4.4.0)
readr_2.1.5 (https://github.com/cran/readr/releases/tag/2.1.5)
stringr_1.5.1 (https://cran.r-project.org/web/packages/stringr/index.html)
dplyr_1.1.4 (https://github.com/cran/dplyr/releases/tag/1.1.4)
ggplot2_3.5.1 (https://cran.r-project.org/web/packages/ggplot2/index.html)
Matrix_1.6-5 (https://cran.r-project.org/src/contrib/Archive/Matrix/Matrix_1.6-5.tar.gz)

For manuscripts utilizing custom algorithms or software that are central to the research but not yet described in published literature, software must be made available to editors and reviewers. We strongly encourage code deposition in a community repository (e.g. GitHub). See the Nature Portfolio guidelines for submitting code & software for further information.

Data

Policy information about availability of data

All manuscripts must include a data availability statement. This statement should provide the following information, where applicable:

- Accession codes, unique identifiers, or web links for publicly available datasets
- A description of any restrictions on data availability
- For clinical datasets or third party data, please ensure that the statement adheres to our policy

The data generated in this study can be downloaded in raw and processed forms from the National Center for Biotechnology Information (NCBI) Gene Expression Omnibus (GEO) under accession numbers GSE208369.

Research involving human participants, their data, or biological material

Policy information about studies with human participants or human data. See also policy information about sex, gender (identity/presentation), and sexual orientation and race, ethnicity and racism.

Reporting on sex and gender	N/A
Reporting on race, ethnicity, or other socially relevant groupings	N/A
Population characteristics	N/A
Recruitment	N/A
Ethics oversight	N/A

Note that full information on the approval of the study protocol must also be provided in the manuscript.

Field-specific reporting

Please select the one below that is the best fit for your research. If you are not sure, read the appropriate sections before making your selection.

☒ Life sciences ☐ Behavioural & social sciences ☐ Ecological, evolutionary & environmental sciences

For a reference copy of the document with all sections, see nature.com/documents/nr-reporting-summary-flat.pdf

Life sciences study design

All studies must disclose on these points even when the disclosure is negative.

Sample size	No statistical methods were used to pre-determine sample sizes but our sample sizes are similar to those reported in previous publications(Veenvliet et al. 2020; Moris et al. 2020, see the references in the main text)
Data exclusions	Sequencing data exclusion criteria is outlined in th method section, including filtering out the substandard data in single-cell measurements, following the general practice in the field.
Replication	Single-cell RNA-seq experiments were done in a single replicate experiment, or two biological replicates for knockout gastruloid Single-cell RNA-seq experiments. All the other experiments were repeated in at least three times.
Randomization	Human conventional gastruloids and RA-gastruloids used in experiments were randomly selected from each timepoint before sample preparation.
Blinding	The Investigators were not blinded to allocation during experiments and outcome assessment.

Reporting for specific materials, systems and methods

We require information from authors about some types of materials, experimental systems and methods used in many studies. Here, indicate whether each material, system or method listed is relevant to your study. If you are not sure if a list item applies to your research, read the appropriate section before selecting a response.

Materials & experimental systems

n/a	Involved in the study
☐	☒ Antibodies
☐	☒ Eukaryotic cell lines
☒	☐ Palaeontology and archaeology
☒	☐ Animals and other organisms
☒	☐ Clinical data
☒	☐ Dual use research of concern
☒	☐ Plants

Methods

n/a	Involved in the study
☒	☐ ChIP-seq
☒	☐ Flow cytometry
☒	☐ MRI-based neuroimaging

Antibodies

Antibodies used

All antibodies are commercially available.
Primary antibody
Goat anti-SOX1 R&D AF3369-SP
Goat anti-SOX2 R&D AF2018
Rabbit anti-SOX2 Millipore AB5603
Rabbit anti-PAX3 ThermoFisher 38-1801
Rabbit anti-PAX3 Thermo 701147
Rabbit anti-PAX8 Proteintech 10336-1-AP
Rabbit anti-WT1 Abcam ab89901
Rabbit Anti-POU3F1 Abcam ab272925
Rabbit Anti-TBX6 Abcam ab38883
Goat Anti-GATA6 R&D AF1700
Rabbit Anti-CDX2 Thermo MA5-14494
Rabbit Anti-TBXT abcam ab209665
Rabbit Anti-SOX10 abcam ab227680

Secondary antibody
Donkey anti-Goat IgG (H+L) Highly Cross-Adsorbed Secondary Antibody, Alexa Fluor Plus 488 ThermoFisher A32814
Donkey anti-Rabbit IgG (H+L) Highly Cross-Adsorbed Secondary Antibody, Alexa Fluor Plus 555 ThermoFisher A32794

Validation

Goat anti-SOX1 R&D AF3369-SP
Validation was performed by the manufacturer using iBJ6 human iPS cells and iBJ6 human iPS cells differentiated into neuroprogenitor cells (https://www.rndsystems.com/products/human-mouse-rat-sox1-antibody_af3369).

Goat anti-SOX2 R&D AF2018
Validation was performed by the manufacturer using D3 mouse embryonic stem cell line, NTera-2 human testicular embryonic carcinoma cell line, F9 mouse teratocarcinoma stem cells, and rat cortical stem cells (https://www.rndsystems.com/products/human-mouse-rat-sox2-antibody_af2018).

Rabbit anti-SOX2 Millipore AB5603
Validation was performed by the manufacturer using Mouse or human embryonic stem cells, and mouse embryonic germ cells (https://www.emdmillipore.com/US/en/product/Anti-Sox2-Antibody,MM_NF-AB5603?ReferrerURL=https%3A%2F%2Fwww.google.com%2F).

Rabbit anti-PAX3 ThermoFisher 38-1801
Validation was performed by the manufacturer using HEK-293, B16-F10, and Neuro-2a cells (https://thermofisher.com/antibody/product/PAX3-Antibody-Polyclonal/38-1801).

Rabbit anti-PAX3 Thermo 701147
Validation was performed by the manufacturer using U2OS, A431, A375, HEK-293, THP1, Mouse Testis and Mouse Cerebellum (https://www.thermofisher.com/antibody/product/PAX3-Antibody-clone-16H22L10-Recombinant-Monoclonal/701147)

Rabbit anti-PAX8 Proteintech 10336-1-AP
Validation was performed by the manufacturer using xxx cells (link).

Rabbit anti-WT1 Abcam ab89901
Validation was performed by the manufacturer using HEK-293T, and K-562 cell lines (https://www.abcam.com/wt1-antibody-ab89901.html).
Rabbit Anti-POU3F1 Abcam ab272925
Validation was performed by the manufacturer using A549 (Human lung carcinoma cells) and mouse fetal brain tissue extracts

(https://www.abcam.com/products/primary-antibodies/oct6-antibody-ab272925.html).

Rabbit Anti-TBX6 Abcam ab38883
Validation was performed by the manufacturer using mouse testis tissue lysate, mouse lung tissue lysate, human testis tissue lysate, and human lung tissue lysate (https://www.abcam.com/products/primary-antibodies/tbx6-antibody-ab38883.html).

Goat Anti-GATA6 R&D AF1700
Validation was performed by the manufacturer using KATO-III human gastric carcinoma cell line and PC-3 human prostate cancer cell line (https://www.rndsystems.com/products/human-gata-6-antibody_af1700).

Rabbit Anti-CDX2 Thermo MA5-14494
Validation was performed by the manufacturer using CaCO2, SW480, HCT116, HeLa and MCF7 (https://www.thermofisher.com/antibody/product/CDX2-Antibody-clone-EPR2764Y-Monoclonal/MA5-14494).

Rabbit Anti-TBXT abcam ab209665
Validation was performed by the manufacturer using Human chordoma tissue, Mouse E14.5 embryo tissue, and Rat E14.5 embryo Tissue cells (https://www.abcam.com/products/primary-antibodies/brachyury--bry-antibody-epr18113-ab209665.html).

Rabbit Anti-SOX10 abcam ab227680
Validation was performed by the manufacturer using Human melanoma tissue; mouse and rat breast tissues (https://www.abcam.com/products/primary-antibodies/sox10-antibody-sp267-ab227680.html).

Donkey anti-Goat IgG (H+L) Highly Cross-Adsorbed Secondary Antibody, Alexa Fluor Plus 488 ThermoFisher A32814
Validation was performed by the manufacturer using LNCaP (positive model) and HeLa (negative model) cells (https://www.thermofisher.com/antibody/product/Donkey-anti-Goat-IgG-H-L-Highly-Cross-Adsorbed-Secondary-Antibody-Polyclonal/A32814).

Donkey anti-Rabbit IgG (H+L) Highly Cross-Adsorbed Secondary Antibody, Alexa Fluor Plus 555 ThermoFisher A32794
Validation was performed by the manufacturer using A549 cells (https://www.thermofisher.com/antibody/product/Donkey-anti-Rabbit-IgG-H-L-Highly-Cross-Adsorbed-Secondary-Antibody-Polyclonal/A32794).

Eukaryotic cell lines

Policy information about cell lines and Sex and Gender in Research

Cell line source(s)	RUES2-GLR were provided by Dr. Ali H. Brivanlou (The Rockefeller University). H9 hESCs were obtained from WiCell. WTC11 hiPSCs were gifted by Dr. Bruce Conklin (Gladstone Institutes).
Authentication	Activities of three knocked-in reporter genes (SOX2-mCit, TBXT-mCer, and SOX17-tdTom) were validated in every experiment with the fluorescence microscope observation. The RUES2-GLR line was authenticated with scRNA-seq.
Mycoplasma contamination	This cell lines are not ested for Mycoplasma contamination.
Commonly misidentified lines (See ICLAC register)	No commonly misidentified cell lines were used.

Plants

Seed stocks	*Report on the source of all seed stocks or other plant material used. If applicable, state the seed stock centre and catalogue number. If plant specimens were collected from the field, describe the collection location, date and sampling procedures.*
Novel plant genotypes	*Describe the methods by which all novel plant genotypes were produced. This includes those generated by transgenic approaches, gene editing, chemical/radiation-based mutagenesis and hybridization. For transgenic lines, describe the transformation method, the number of independent lines analyzed and the generation upon which experiments were performed. For gene-edited lines, describe the editor used, the endogenous sequence targeted for editing, the targeting guide RNA sequence (if applicable) and how the editor was applied.*
Authentication	*Describe any authentication procedures for each seed stock used or novel genotype generated. Describe any experiments used to assess the effect of a mutation and, where applicable, how potential secondary effects (e.g. second site T-DNA insertions, mosiacism, off-target gene editing) were examined.*
